# Development of Electromagnetic Shielding Composites Reinforced with Nonwovens Produced from Recycled Fibers

**DOI:** 10.3390/polym15224469

**Published:** 2023-11-20

**Authors:** Melisa Atay, Deniz Duran Kaya, Aydın Ülker

**Affiliations:** 1 Graduate School of Natural and Applied Sciences, Ege University, 35100 Izmir, Turkey; melisa_atay@hotmail.com; 2 Department of Textile Engineering, Faculty of Engineering, Ege University, 35100 Izmir, Turkey; 3 Department of Mechanical Engineering, Faculty of Engineering and Architecture, İzmir Katip Çelebi University, 35620 Izmir, Turkey; aydin.ulker@ikcu.edu.tr

**Keywords:** electromagnetic shielding, electrical conductivity, nonwoven, recycling, composite

## Abstract

As a light-weight solution for electromagnetic shielding, this paper aims to investigate the development of electrically conductive composites that shield from electromagnetic radiation while providing sustainability by using recycled fibers in the structure of nonwoven reinforcement materials. The main novelty of this research is the conversion of waste fabrics into functional composites via a fast and inexpensive method. For this purpose, waste fabrics were recycled into fibers, and the recycled fibers were processed into needle-punched nonwovens to be used as reinforcement materials for electromagnetic shielding composites. Electrically conductive composite structures were obtained by adding copper (II) sulfate and graphite conductive particles with different ratios to polyester resin. The hand lay-up method was used for the production of composites. Electromagnetic shielding, electrical resistivity, and some mechanical properties of the composites were investigated. The results were analyzed statistically using IBM SPSS software version 18. The results have shown that up to 31.43 dB of electromagnetic shielding effectiveness was obtained in the 1–6 GHz frequency range. This result corresponds to a very good grade for general use and a moderate grade for professional use, according to FTTS-FA-003, exceeding the acceptable range for industrial and commercial applications of 20 dB. The composites developed in this research are good candidates to be used in various general and professional applications, such as plastic parts in household applications, electronic industry, building and construction industries, and other applications where light weight shielding materials are needed.

## 1. Introduction

With the rapid growth of electrical and electronic devices and accessories, which emit electromagnetic (EM) energy in different frequency bands, exposure to EM radiation has increased [1]. The expansion of the electronic industry and the extensive use of electronic equipment in communications, computations, automation, biomedicine, space, and other purposes have led to problems such as electromagnetic interference of electronic devices and health issues [2].

The performance and operation of electronic and electrical equipment, as well as the health of both active and passive users, are all hampered by electromagnetic radiation. The issue of electromagnetic shielding interference is getting worse due to the shrinking of electronic gadgets and the development of wireless technologies [3]. Increasing electromagnetic wave pollution induced by the use of technology today causes harm to the health of humans and other living beings and to nature. In order to minimize the electromagnetic damage, both from the point of view of the health of humans and the environment, investigations into electromagnetic shielding have recently become more significant [4].

Electromagnetic shielding effectiveness is achieved by either conductive or magnetic materials in the structure. Conventional textile materials are not electrical conductors, but they can be turned into electrical conductors by using several methods, such as adding conductive particles in different forms, coating, using conductive polymers, etc. Most metals in different forms are added to the structure to obtain electromagnetic shielding effectiveness. On one hand, metals give the highest electromagnetic shielding effectiveness to the structure they are added to, due to their high electrical conductivity, but on the other hand, metals have some disadvantages such as heaviness, stiffness, corrosion, the difficulty of being processed with conventional textile machinery, and high cost [5]. Textile-reinforced composite structures also have an important role in electromagnetic shielding [3]. Given their potential to reduce unwanted radiation and other special features, composites are highly advised for use as shielding materials. Their light weight, flexibility, durability, and low price are equally crucial in this context as in their radiation shielding qualities. Polyester-based composites are among the most favored materials because of their exceptional qualities [6].

Recently, a lot of research has been carried out to investigate the electromagnetic shielding effectiveness of conductive polymeric composites, and there is much research in this field.

Liu et al. (2017) synthesized two novel flowerlike NiO hierarchical structures, roseflower and silkflower, by using a facial hydrothermal method coupled with a subsequent postannealing process. The structures, morphologies, and magnetic and electromagnetic properties of two NiO structures have been systematically investigated. They found that, because of their large surface-to-volume ratio and hierarchical structures, the NiO nanoflowers exhibited strong absorbing performance, which could be considered a new generation of absorption materials [7].

Nan et al. (2023), outlined the advances in low-dimensional EMI shielding nanomaterial-based elastomers and addressed electromagnetic shielding elastomers as the future of electromagnetic shielding materials. Approximately 45–55 dB shielding effectiveness was obtained with AgNPs/SEBS, 32–106 dB was obtained with AgNWs with different matrices, 35–54.8 dB was obtained with CNTs with different matrices, >75 dB was obtained with LMs with different matrices, 60 dB was obtained with PEDOT:PPS, and 20–80 dB was obtained with graphene-based composites [8].

Fan Z. et al. (2021), used polyvinylidine fluoride (PVDF) as an adhesive for electromagnetic shielding, glued a non-woven surface with graphene (GE) nanosheets, and obtained a composite. They found that the composite produced has a shielding ability of 20 decibels between 1 and 18 GHz [9].

Lu et al. (2017) produced a composite non-woven surface called CEF-NF using bicomponent fibers, including carbon fiber (CFS), polypropylene core, and polyethylene sheath. They noted that the shielding ability can reach 40%, but the shielding ability increases pareto with long fibers in the composite [10].

Wong et al., (2010) described the development of an electromagnetic interference shielding material using recycled carbon fiber. Fiber recycled from a fluidized bed process was transformed into a non-woven veil and molded into a glass-fiber-reinforced polymer plaque to provide shielding. The performance of shielding was measured using virgin fiber, and the results were compared to a recycled fiber veil. With increasing veil areal densities, shielding effectiveness improved [11].

Ali et al., (2022) studied the utilization of waste resources (i.e., carbon particles and Kevlar fabrics) to create hybrid composites with higher electrical properties coupled with high mechanical strength. The composite sample with 8 g of carbon particles led to the lowest electrical resistivity, EMI shielding (35.6 dB), and excellent mechanical performance [12].

Due to their vast application potential, conductive polymer composites have garnered a great deal of attention. A significant use of conductive polymer composites is as electromagnetic shielding materials because of their high mechanical deformation capacity, controlled conductive characteristics, and process efficiency [13].

Carbon is one of these polymers. The scientific world has recently become more interested in carbon because of its superior mechanical, thermal, and electrical qualities [14]. Graphite is a versatile industrial mineral with unique properties that have facilitated technological innovation. Today, natural graphite is a key component in high-performance refractory linings for steel manufacture, high-charge capacity anodes for lithium-ion batteries, and a source of graphene to inspire a new generation of smart materials. Graphite is a crystalline form and a natural allotrope of carbon. In graphite, carbon atoms are bonded to three other carbon atoms to form strong, two-dimensional layers that are extremely stable, but each layer is only weakly linked to adjacent layers by van der Waal’s forces. The resulting hexagonal-layered structure forms one of the softest minerals. The presence of an unpaired valence electron makes graphite an excellent electrical conductor within the plane of the layers. Also, graphite is inert towards most chemicals and has a high melting point of ~3550 °C [15].

Copper (II) sulfate, also known as copper sulfate, is an inorganic compound with the chemical formula CuSO_4_. Copper sulfate/sulfuric acid solutions form the basis of electrolytes [16].

For use as reinforcement in polymer composites, needle-punched nonwoven textiles offer a number of benefits, such as strong z-directional strength that considerably lowers delamination issues. The high void volume composition of nonwoven textiles also makes it easy for the fabrics to absorb resin, and thick parts can be made affordably. These textiles’ compressibility has the added benefit of making various shapes simple to create. The composites are also often made stronger and tighter during the reinforcing step [17,18,19].

Cheng et al. (2022) mentioned that with the rapid advances in flexible and wearable electronics, the corresponding EMI shielding materials should also possess low density (i.e., lightweight), high thermal stability, appreciable mechanical fexibility, and corrosion resistance, in addition to effective EMI shielding performance, and stated that the currently developed flexible EMI shielding materials are mainly based on carbon materials, polymers, and MXene-based materials [20].

Nonwoven textiles exhibit a variety of, often hardly understandable, behaviors due to their anisotropic structure as needled nonwoven materials. Needle punching is a purely mechanical, environmentally friendly, and economical process [21]. The use of nonwovens as EMI shielding materials is potentially favorable due to their porous structure, controllable thickness, low cost, and high flexibility [22]. Nonwovens are porous materials. Nan et. al. (2023) mentioned that the inner pore structure variation of the material also impacts the EMI loss power, including multiple resonances, in addition to the material’s macroscale electrical and geometric properties [8]. Zeng et al. (2017) investigated the intrinsic shielding mechanism of the porous materials and demonstrated that compression reduces the pores and, in turn, the multiple refractions [23]. Conductive nonwovens capable of providing EMI shielding can be developed by various methods, such as applying conductive surface treatments, blending with conductive fibers, or adding conductive particles to the structure. Pakdel et al. (2021) achieved 85 dB electromagnetic shielding effectiveness with nonwovens produced from carbon fiber wastes and polyamide by the needle punching method [22]. Lu et al. (2017) achieved 30.29 dB of electromagnetic shielding effectiveness with CEF-NF composites produced via two-step wet-papermaking/thermal-bonding processes [10].

In general, the manufacture of nonwovens requires two main steps, namely web formation and web bonding. In the web formation, the fibers are laid on a forming surface that can be either dry, airtight, wet, or spun. The fibers are transformed into continuous layers of loosely arranged webs or networks. In general, the formed web exhibits poor physical properties; therefore, web bonding is required to achieve the needed cohesion between fibers. Mechanical, thermal, or chemical systems can be used for web bonding. Among the production methods of nonwovens, the needle-punching method is suitable for the production of complex net-shape and near-net-shape preforms. In addition, needle-punched nonwoven natural fibers have excellent through-thickness properties that reduce delamination problems [24,25,26]. During the needling process, fibers in the felt are joined to other fibers in the felt using needles with branched characteristics that transfer fibers from the felt’s surface to its depth. By doing so, a product with a unique structure that is resistant to mechanical action is created, and it finds numerous and broad applications [18].

Some of the related studies about electromagnetic composites produced with nonwoven reinforcement in the literature and their main differences from our paper are given in Table 1.

Even though there is much research about electromagnetic shielding composites, there are fewer studies about nonwoven reinforced composites produced from recycled fibers, as can be seen in Table 1. This paper fills the gap by investigating the effects of material properties on the electromagnetic shielding of needle-punch nonwoven reinforced composites made from recycled fibers. Also, most of the studies in the literature focus on production methods like coating, laminating, etc., or include expensive fibers. (See Table 1). In this paper, the easy, low-cost, and environmentally friendly production method of needle punching was chosen for the production of nonwoven reinforcement material.

Recycling is a way to recycle waste into new products, which is used to prevent the waste of potentially useful materials. The importance of this study is that garment waste was recycled and used for the development of a new and functional product structure. This way, textile wastes were brought back to the circular economy as high-value-added products, and the production and study of the properties of electromagnetic shielding composites, which are functional and high-value-added materials, were also achieved [21].

## 2. Materials and Methods

In this study, textile waste was recycled to obtain recycled nonwoven reinforcement materials, and the nonwovens produced were used in the production of electromagnetic shielding composites. Electromagnetic shielding, electrical resistivity, and mechanical properties of the developed composites were investigated.

### 2.1. Recycling Process

**Figure 1:**
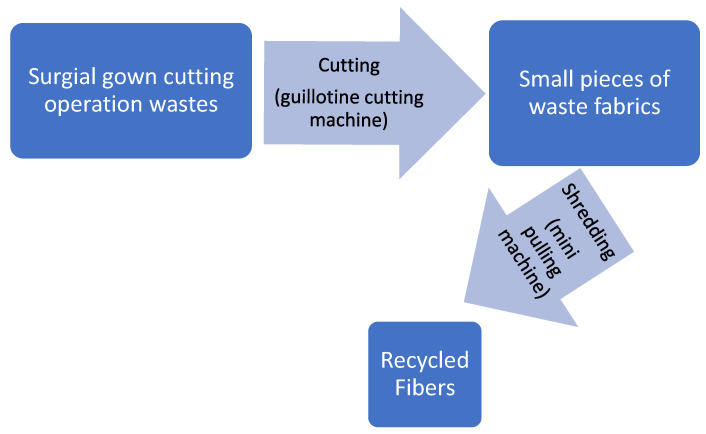
Flow diagram of the recycling process.

Wastes from the apparel production, namely the cutting operation of surgical gowns, were chosen as textile waste to be recycled. The waste was obtained from the Ege University Textile Engineering Department’s apparel production training mill. Surgical gown wastes were made of 100% cotton in plain weave fabric construction. In the recycling process, garment waste from surgical gowns were turned into waste fibers. A flow diagram of the recycling process can be seen in Figure 1.

**Figure 2:**
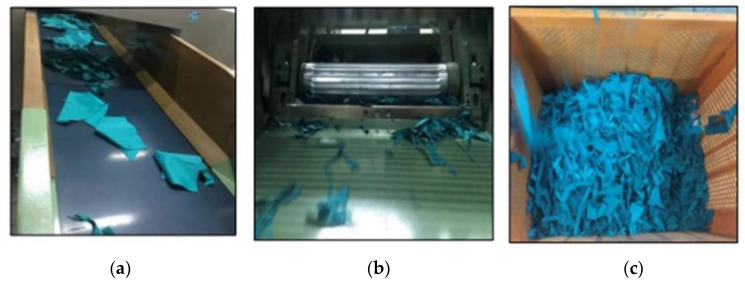
Pictures from the cutting process: (**a**) Waste fabrics before the cutting process on the Balkan DT61 guillotine cutting machine; (**b**) Blade cutting the waste fabrics in the guillotine cutting machine; (**c**) Small pieces of waste fabrics after the cutting process [19].

In the first stage of the recycling process, the surgical gowns were cut into smaller pieces (about 5 cm) in two passages. by using the DT61 guillotine cutting machine, from Balkan Textile Machinery, Aydın, Turkey. The cutting speed of the machine was 175 rpm. Pictures from the cutting operation are given in Figure 2 [19].

The second stage of the recycling process was the shredding process. In this process, pieces of the waste fabrics were processed in a Balkan DT10 mini-pulling machine with three section cylinders to be turned into recycled fibers. Recycled fibers were obtained after 3 passages, which means the textile wastes have been passed through the mini-pulling machine 3 times. The speed of the section cylinders of the pulling machine (m/min) was as follows: 2.75 for the first section, 3.34 for the second, and 3.50 for the third section. The Balkan DT10 mini-pulling machine is mainly for small-scale production. Woven, knitted scraps, and other scraps can be processed by this machine. The first neck cylinder is steel wire, and the other neck cylinders are rubber rollers. The cylinder with pin and feeder tray setting (for 1. cylinder 20 mm, for 2. cylinder 43 mm, for 3. cylinder 24 mm) and the waste knife flap setting (for 1. cylinder 2, for 2. cylinder 5, for 3. cylinder 2) have been fixed for all passages [37].

**Figure 3:**
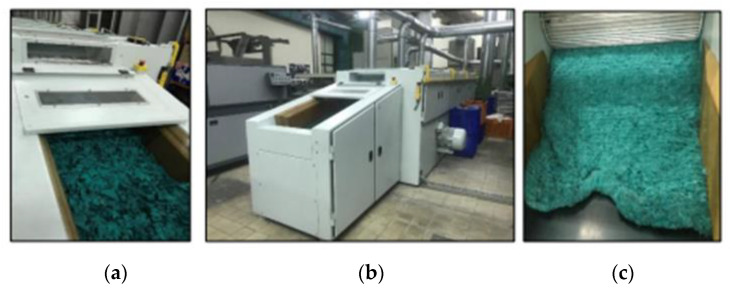
Pictures from the defibrillation process: (**a**) Small pieces of waste fabrics before the defibrillation process on the rug pulling machine; (**b**) Rug pulling machine; (**c**) Waste fibers as output of the defibrillation process [21].

Pictures from the defibrillation process are given in Figure 3 [21].

### 2.2. Nonwoven Production

**Figure 4:**
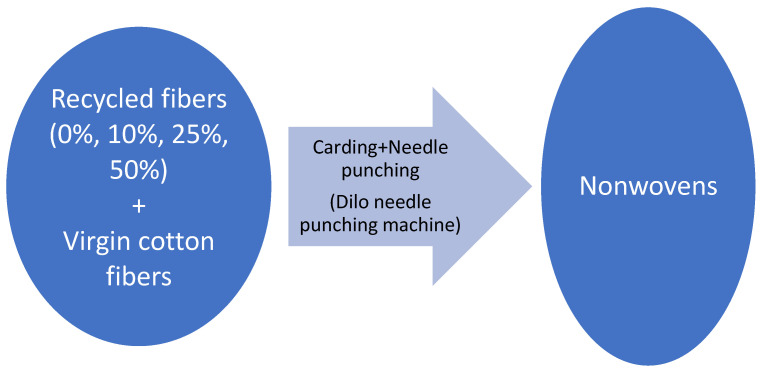
Flow diagram of the nonwoven production process.

Nonwoven reinforcement materials were obtained by carding and needle punching technology using recycled fibers obtained by cutting and shredding processes. It is a dry-laid process starting with carding, during which the small tufts are separated into individual fibers that are bound together, parallelized, and delivered in the form of a web [24]. A flow diagram of the nonwoven production process is given in Figure 4.

Groove needles are used for fiber entanglement and thus bonding. A laboratory size Dilo needle punching machine was used for the production of nonwovens in this paper. This machine consists of one carding, one overlapping, and one needle punching section, and it is suitable to be used in small factories or training mills. In the actual production in normal-sized factories, there are bigger and much more developed machines with several carding, overlapping, and needle punching sections, automated systems, and control units to ensure quality.

**Figure 5:**
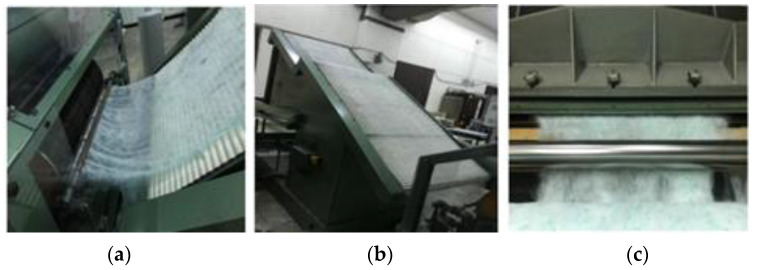
Pictures from nonwoven production on a laboratory-size Dilo needle punching machine: (**a**) Fibrous web leaving the card; (**b**) Carded web on the cross-lapper; (**c**) Bonded nonwoven leaving the needling section [21].

Pictures from the nonwoven production are given in Figure 5 [21].

Needle punching was chosen since it is a purely mechanical and environmentally friendly method that enables the production of voluminous nonwoven structures as suitable materials for composite reinforcement with voids in their structure [19]. During the production process, fibers obtained by the recycling process were blended with virgin cotton fibers in different ratios (0%, 10%, 25%, and 50%) and turned into nonwoven structures via carding and needle punching processes. In practical applications, recycled fibers are blended with virgin fibers for processability since fiber length and mechanical properties are reduced during cutting and shredding operations. Various blend ratios (20%, 40%, 60%, 50%, 80%…) were investigated in similar studies in the literature [19,22].

The blend ratios in this paper were chosen as 0, low, medium, and high in order to compare and investigate the effect of waste fiber ratio, with 50% being the highest ratio of waste fibers that could be processed with the needle punching machine used in this research. While choosing the blend ratios, processability and experience from the previous research were considered. Optimal blending ratios can be chosen during actual production in normal-sized factories [19]. The process parameters of the needle punching are given in Table 2 [21].

### 2.3. Composite Production

In this study, composites reinforced with nonwoven textiles produced from recycled fibers were developed for electromagnetic shielding applications. Polyester (PES) resin (1.1 g/cm^3^), together with 6% Cobalt Octobalt (0.92–0.97 g/cm^3^) as speeder and methyl ethyl ketone peroxide (1.16 g/cm^3^) as stiffener, was used as the matrix of the composite. PES resin was obtained from a local supplier. The commercial code of the resin is YCL MKP-60. From the resin supplier’s recommendation and based on our experiments (pre-trials) in the laboratory before production, the ratios of the stiffener and speeder in the PES matrix were chosen as 0.7% and 0.5%, respectively.

Conductive fillings, namely graphite (25 micron particle size) and copper sulfate (CuSO_4_) (1–3 mm particle size) powders, were added to the resin of the composites to provide electromagnetic shielding. Conductive fillings were added in different ratios, namely 0%, 10%, and 30%.

Composites were produced by the hand-lay-up method. For the production of composites, first of all, a liquid release agent was applied to the surface of the glass mold via a sponge to separate the composites from the glass after the production. Firstly, polyester resin without conductive fillers was applied to the nonwoven reinforcement materials with a brush. Then, polyester resin was blended with copper (II) sulfate and graphite powders in the mentioned ratios, respectively, for comparison. Polyester resins, including copper (II) sulfate and graphite powders, were applied to the nonwoven reinforcement materials by the same method. There are various fiber-to-rein ratios (20%, 30%, 40%, 50%, 60%) in the literature [38]. In this study, the fiber-to-rein ratio was chosen at 25% based on the prior experiments in the laboratory. After 24 h of curing, the composites were cut into pieces in accordance with test specifications. Table 3 shows the matrix of nonwoven reinforced composite samples.

### 2.4. Testing

Thickness, basis weight, electromagnetic shielding effectiveness, electrical resistivity, tensile strength, and bending strength tests were applied to the composites. The results were evaluated statistically using the SPSS software program. Analysis of variance (ANOVA) tests and SNK (Student–Neuman–Kleus) tests for subgroup analyses were performed at a 95% confidence interval.

Thickness and basis weight tests were applied according to ASTM D 7291 and ASTM D 792 standards, respectively [39,40].

#### 2.4.1. Electromagnetic Shielding Tests

Electromagnetic shielding effectiveness tests were performed according to the EN50147-1 standard in the frequency range of 1 GHz to 6 GHz using an anechoic chamber test system [1,5,41].

**Figure 6:**
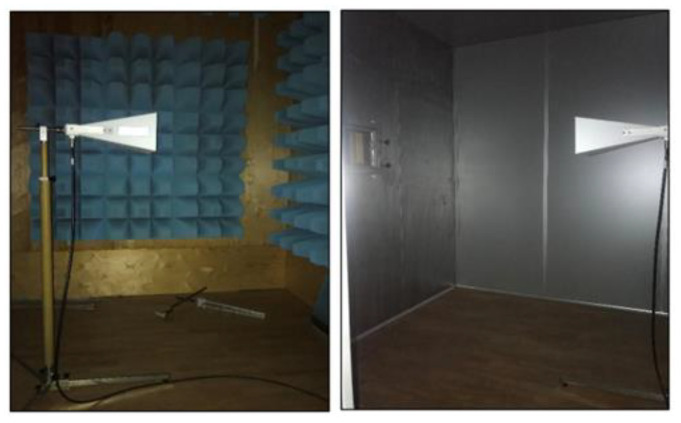
Shielded rooms with transmitting and receiving antennas [21].

During the measurements, the sample was placed between a signal generator and a receiver. The signal produced in the signal generator was first amplified and then sent onto the sample through the transmitting antenna. A part of the radiation was blocked by the sample, and the remaining was transmitted through the sample to the receiving antenna [1,5]. Shielded rooms with transmitting and receiving antennas are shown in Figure 6.

According to the test principle, the value of the electromagnetic field blocked by the sample is calculated by measuring the value of the signals transmitted to the other side by the sample placed between the signal generator and the receiving antenna. The measurement value of the EM wave was expressed in dBmV, and then the reduction of the EM wave was obtained from the difference of the two measurements in dB [1,5,41,42].

#### 2.4.2. Electrical Resistivity Tests

Electrical resistance measurements were performed using the Keithley 65 Electrical Resistivity Test Fixture which was sourced from Art Electronic Systems, Ankara, Turkey.

**Figure 7:**
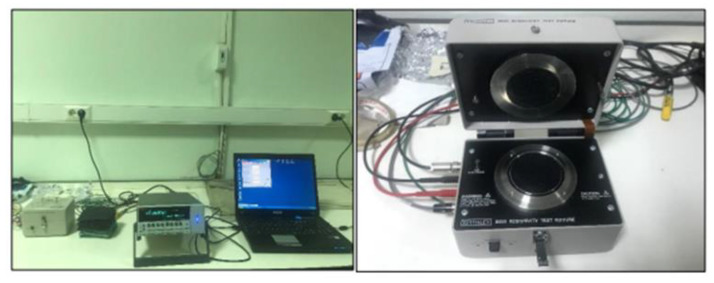
Pictures of an electrical resistivity test fixture [21].

During the measurement, the fabric is clamped between two electrodes, and the appropriate voltage is applied to the fabric. The current created by this voltage in the fabric is measured by the system, and the electrical resistance of the fabric is calculated by the system using Ohm’s Law (R = V/I). Pictures of the test fixture are shown in Figure 7.

Measurements were made according to the surface resistance measurement principle defined in the ASTM D 257 standard, using a 500 ± V voltage and a 60 s electrification time [43].

Surface resistivity (r) was described as the resistance to leakage current along the surface of an insulating material, and it was reported in ohms per square centimeter (ohm/cm^2^). The electrical resistivity was measured between two parallel electrodes in contact with the specimen surface and separated by a distance equal to the contact length of the electrodes. Since the surface length is fixed during the measurement, the measurement takes place independently of the physical dimensions (thickness and surface area) of the sample. Surface resistivity is expressed in ohms per unit area. As the surface resistivity of the material increases, its electrical conductivity decreases.

Volume resistance, unlike surface resistance, is expressed as the number of ohms per unit volume. Therefore, thickness is a factor that affects volume resistance. Volume resistivity is expressed as the number of ohms per unit volume (ohm × cm) [5,43].

#### 2.4.3. Tensile Tests

**Figure 8:**
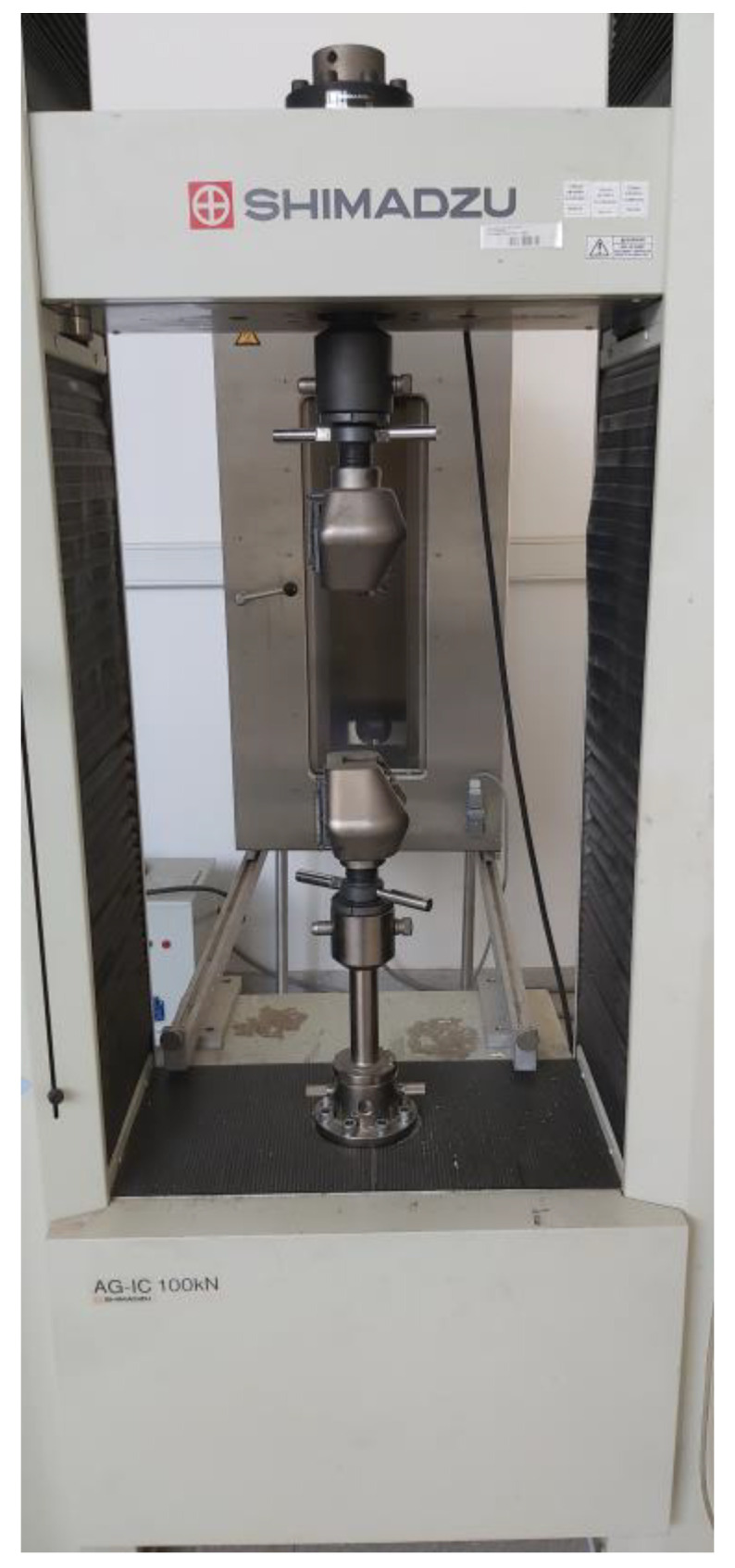
SHIMADZU Universal Testing Machine [21].

The tensile test samples were prepared to be 250 mm in length and 25 mm in width based on tensile specimen geometry recommendations in ASTM D3039 [44], and then the samples were tested in a SHIMADZU AGS-X universal testing machine (Figure 8) with 100 kN of load cell at a constant crosshead speed of 2 mm/min. The load was set to 50 kN. Six samples were used for each type for the tensile tests.

The displacement and breaking force were recorded during the tests to evaluate the mechanical performances of the samples [44].

#### 2.4.4. Flexural Tests

The flexural test samples were cut at 125 mm for length and 25 mm for width, and then the three-point tests were performed in the SHIMADZU AGS-X universal testing machine by adjusting fixtures with a support span of 32 mm and a deformation rate of 2 mm/min in accordance with ASTM D790 [45]. Six samples were used for each type for the flexural tests [45].

## 3. Results and Discussion

### 3.1. Results of Thickness Measurements

**Figure 9:**
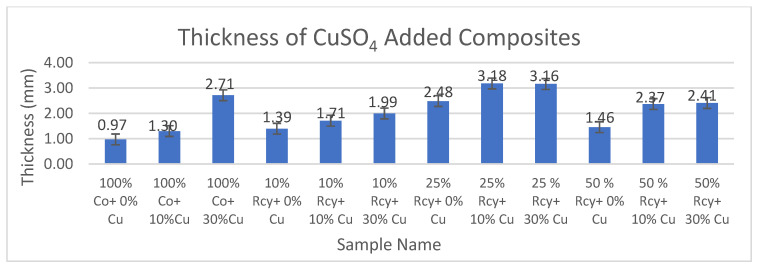
Average thickness results of CuSO_4_ added composites.

**Figure 10:**
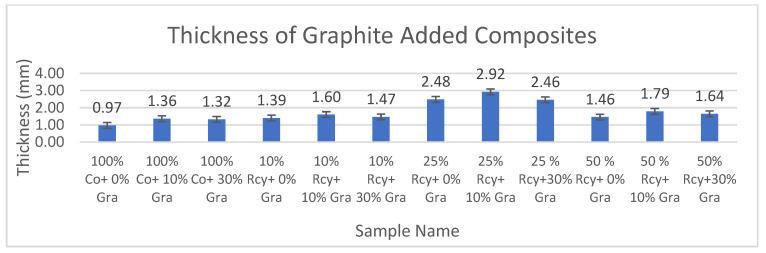
Average thickness results of graphite added nonwoven reinforced composites.

The results of thickness measurements of CuSO_4_ and graphite-added composites are given in Figure 9 and Figure 10, respectively.

As it is observed from Figure 9, the thickness values of composites increase with increasing ratios of particles in the structures. The thickness values of the conductive composites developed were in the range of 1.30–3.18 mm, which are quite thin compared to other electromagnetic shielding materials.

### 3.2. Results of Basis Weight Measurements

**Figure 11:**
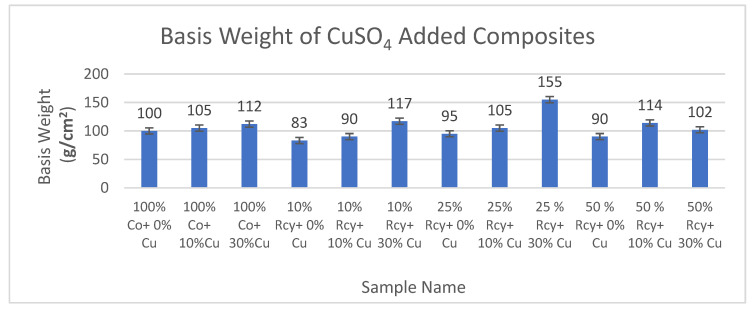
Average basis weight results of CuSO_4_ added composites.

**Figure 12:**
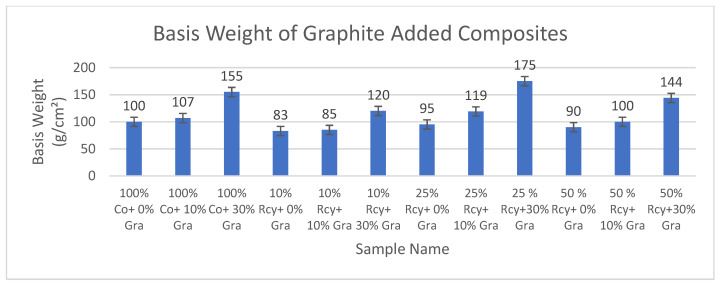
Average basis weight results of graphite-added nonwoven reinforced composites.

The results of basis weight measurements of CuSO_4_ and graphite added composites are given in Figure 11 and Figure 12, respectively.

Results have shown that the basis weight of composites increases with increasing ratios of particles in the structure. The basis weight of the composites developed in this study was found to be between 83 and 175 g/m^2^. Therefore, it is observed that light electromagnetic shielding materials were developed.

### 3.3. Results of Electromagnetic Shielding Measurements

**Figure 13:**
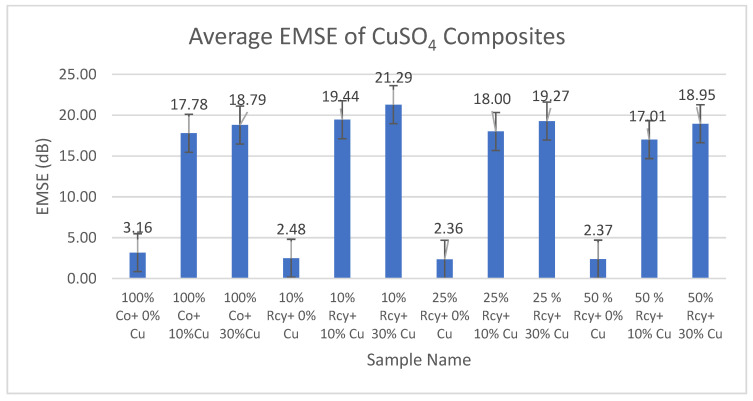
Average EMSE results of CuSO_4_ added composites.

**Figure 14:**
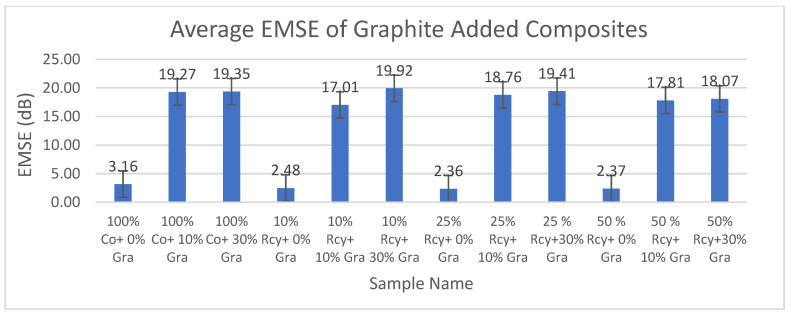
Average EMSE results of graphite added composites.

Results of electromagnetic shielding effectiveness tests of the CuSO_4_ added composites are given in Figure 13, and results of graphite-added composites are given in Figure 14. Results of ANOVA tests have shown that CuSO_4_ ratio, graphite ratio, thickness, basis weight, surface and volume resistivity, and frequency have statistically significant effects on the EMSE of nonwoven reinforced composites in a 95% confidence interval.

It can be noted that, Electromagnetic Shielding Effectiveness (EMSE) increases with an increasing CuSO_4_ ratio in the structure. This is because of the increased electrical conductivity due to the increase of conductive particles in the structure. EMSE results for 30% CuSO_4_ added samples were found to be higher than those for 10% CuSO_4_ added samples. Among the CuSO_4_ added samples, the lowest EMSE value was seen at 1 GHz, at 5.21 dB, with the sample including 10% recycled fibers and 10% CuSO_4_. The highest EMISE value, 35.87 dB, was seen at 6 GHz with the sample including 10% recycled fibers and 10% CuSO_4_ inclusion.

SPSS analysis results have shown that the effect of composite material thickness and basis weight on EMSE is statistically significant. It was observed that the EMSE value increased with increasing thickness and basis weight of composites. This can be explained by the increase in the amount of conductive particles in the structure as the weight increases and also by the increase in attenuation caused by internal reflections throughout the thickness [8]. The effects of surface resistivity and volume resistivity of nonwoven reinforced composites on EMSE were found to be statistically significant. Liu et al. (2021) mentioned that conductivity also becomes the most important component in determining the EMI shielding performance when it varies dramatically and exponentially in comparison with the thickness [8,46]. For electrically conductive shielding materials, electromagnetic shielding effectiveness increases with increasing electrical conductivity [1]. It is a well-known fact that conductivity is the inverse of resistivity [47]. Therefore, electromagnetic shielding effectiveness increases as resistivity decreases.

**Figure 15:**
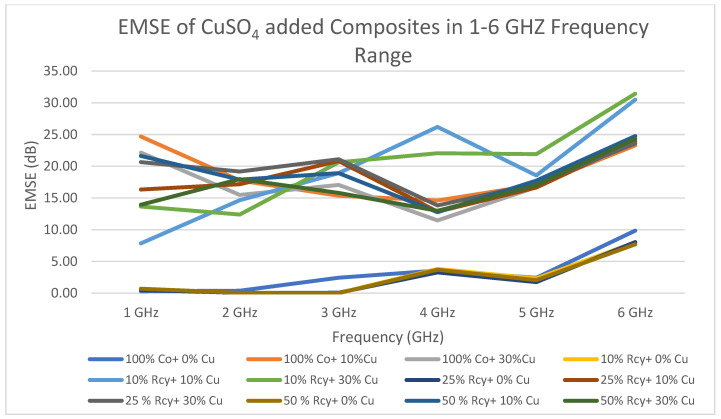
Change in EMSE of CuSO_4_ added nonwoven reinforced composites in the 1–6 GHz frequency range.

**Figure 16:**
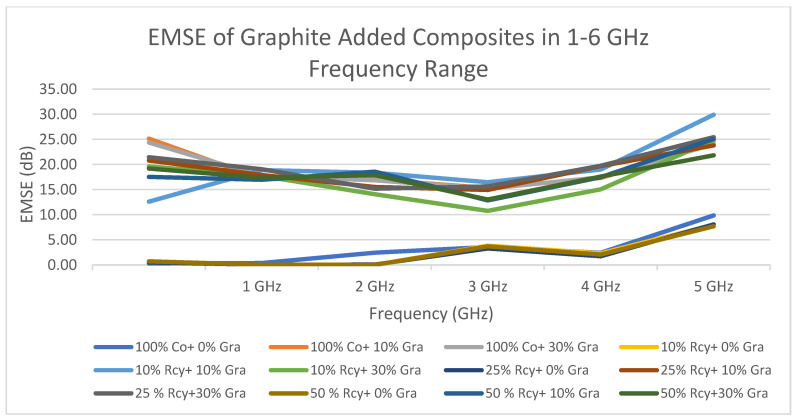
Change in EMSE of graphite-added nonwoven reinforced composites in the 1–6 GHz frequency range.

The effect of frequency of nonwoven reinforced composites on electromagnetic shielding effectiveness was found to be statistically significant. The changes in EMSE of composites by frequency are shown in Figure 15 and Figure 16. It can be seen that the electromagnetic shielding effectiveness values of the composites increased with increasing frequency. In other words, generally, the highest electromagnetic shielding effectiveness value has been obtained at 6 GHz. The lowest average electromagnetic shielding effectiveness value was obtained at 14.39 dB at 2 GHz, and the highest average electromagnetic shielding effectiveness value was obtained at 26.21 dB at 6 GHz.

The highest EMSE results were obtained at 26.21 dB at 6 GHz with graphite-added composites and 35.87 dB at 6 GHz with CuSO_4_ added composites. In the literature, there are studies that achieved higher EMSE results with nonwoven reinforced composites. But most of them do not include recycled fibers, are produced by methods like coating, laminating, etc., or include expensive fibers. (See Table 1). One of the advantages of the composites developed in this paper is the easy, low-cost, and environmentally friendly production method of the reinforcement material.

### 3.4. Results of Electrical Resistivity Measurements

#### 3.4.1. Surface Resistivity

Results of ANOVA tests have shown that CuSO_4_ ratio, graphite ratio, thickness, and basis weight have statistically significant effects on the surface resistivity of nonwoven reinforced composites, within a 95% confidence interval.

**Figure 17:**
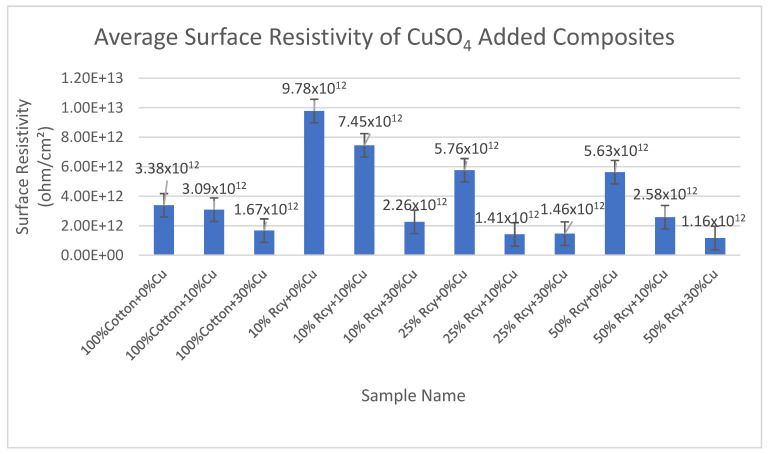
Average surface resistivity of CuSO_4_ added composites.

**Figure 18:**
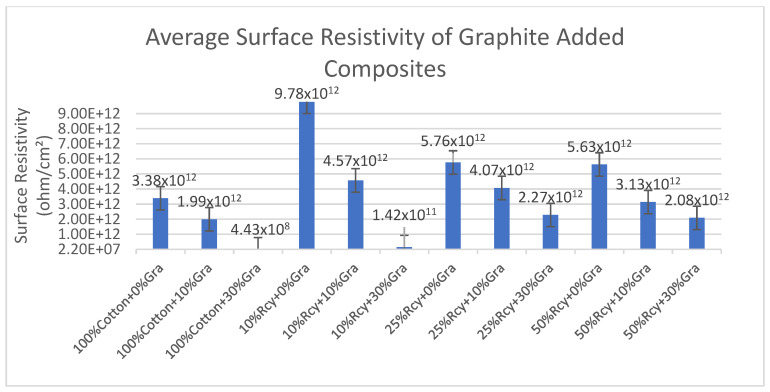
Average surface resistivity of graphite added composites.

Results of surface resistivity tests of the CuSO_4_ added nonwoven reinforced composites are given in Figure 17, and results of graphite-added nonwoven reinforced composites are given in Figure 18.

It is seen in Figure 17 that the effect of conductive particle inclusion on surface resistivity is that 30% CuSO_4_ including composites exhibited lower surface resistivity results compared to 10% and 0% CuSO_4_ including composites. The lowest surface resistivity results were obtained at 1.16 × 10^12^, with 50% recycled fiber and 30% CuSO_4_ included in the sample. This is due to the higher CuSO_4_ amount in the structure, thus the higher electrical conductivity. The same trend was valid for graphite, including samples. In general, it is observed that surface resistivity decreases with an increasing amount of conductive particles in the structure.

The effect of the basis weight and thickness of the composite on surface resistivity was statistically significant. Higher thickness and basis weight resulted in a higher amount of CuSO_4_ in the structure and thus higher conductivity. The lowest surface resistivity results were obtained from 100% cotton and 30% graphite, including the sample, at 4.43 × 10^8^ ohm/cm^2^, among the graphite-added composites. Surface resistivity decreased with increasing thickness and basis weight.

#### 3.4.2. Volume Resistivity

Results of ANOVA tests have shown that waste fiber ratio, CuSO_4_ ratio, graphite ratio, thickness, and basis weight have statistically significant effects on the surface resistivity of nonwoven reinforced composites, within a 95% confidence interval.

**Figure 19:**
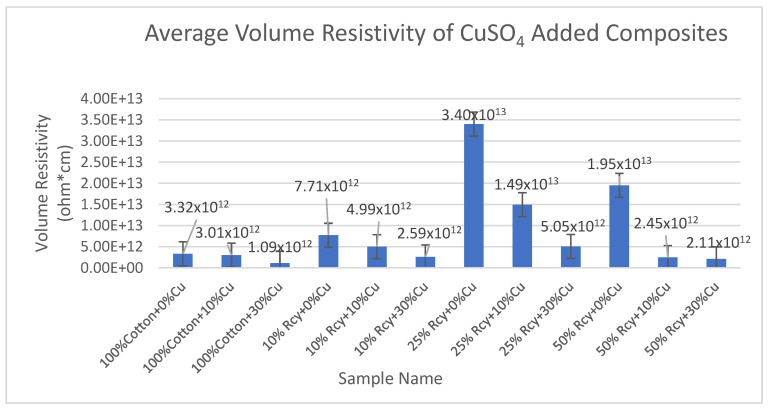
Average volume resistivity of CuSO_4_ added nonwoven reinforced composites.

**Figure 20:**
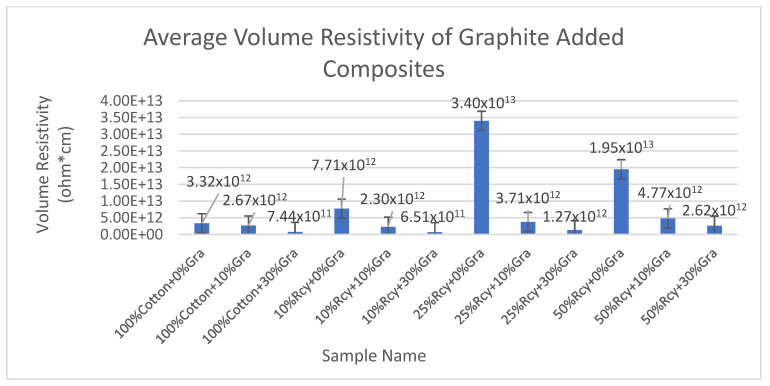
Average volume resistivity of graphite-added nonwoven reinforced composites.

Results of volume resistivity tests of the CuSO_4_ added nonwoven reinforced composites are given in Figure 19, and results of graphite-added nonwoven reinforced composites are given in Figure 20.

In general, it can be deduced that an increase in waste fiber ratio results in an increase in electrical volume resistivity due to decreasing thickness. This can be explained by the higher amount of conductive particles and, thus, higher conductivity in thicker structures.

It is observed that samples that do not contain graphite exhibit the highest volume resistivity results. Although the difference between the results of 10% and 30% graphite-containing samples was not statistically significant, 30% graphite-containing composites had the lowest volume resistivity value. The lowest volume resistivity value was obtained from 10% recycled fiber and 30% graphite, including the sample, as 6.51 × 10^11^ ohm × cm, among the graphite-added composites. This is an expected result due to the higher conductive particle amount in the structure.

As for the composites with different CuSO_4_ ratios, it is observed that the lowest volume resistivity results were obtained from 30% CuSO_4_ containing samples. The lowest volume resistivity value was obtained from 100% cotton and 30% CuSO_4_ samples, as 1.09 × 10^12^ ohm × cm, among the CuSO_4_ added composites.

Even though the difference between the results of 0% and 10% CuSO_4_ containing samples is statistically not significant, 10% CuSO_4_ containing samples had lower volume resistivity results compared to 0% CuSO_4_ containing samples.

In general, it is concluded that volume resistivity decreased with an increasing CuSO_4_ ratio in the structure due to higher conductivity, as expected.

The effect of thickness and basis weight of nonwoven reinforced composites on volume resistivity was found to be statistically significant. The volume resistance increased as the thickness decreased. This is due to the lower amount of resin and conductive fillers when the thickness and basis weight of nonwoven reinforced composites decrease.

### 3.5. Results of Tensile Tests

#### 3.5.1. Tensile Strength

Results of ANOVA tests have shown that waste fiber ratio, CuSO_4_ ratio, thickness, basis weight, reinforcement material’s thickness, reinforcement material’s basis weight, and reinforcement material’s air permeability have statistically significant effects on the tensile strength of composites within a 95% confidence interval.

**Figure 21:**
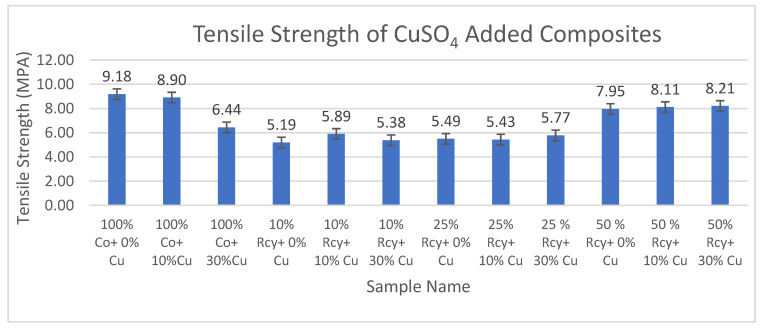
Tensile strength values of CuSO_4_ added composites.

**Figure 22:**
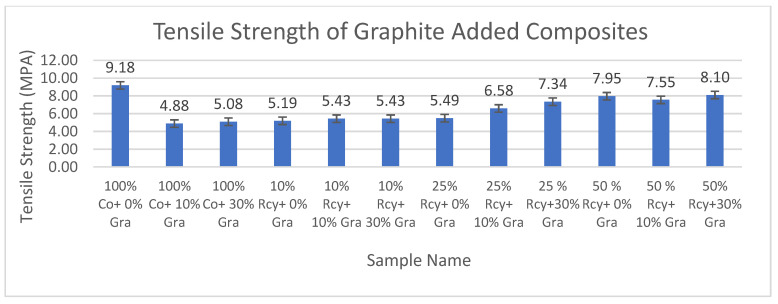
Average tensile max. strength values of graphite-added composites.

Results of tensile strength tests of the CuSO_4_ added nonwoven reinforced composites are given in Figure 21, and results of graphite-added nonwoven reinforced composites are given in Figure 22.

Although the results of tensile strength are close to each other, the addition of CuSO_4_ caused a decrease in the tensile strength of composites. This can be attributed to the agglomeration effect caused by the addition of CuSO_4_. The highest tensile strength results were obtained with a 100% cotton sample that did not include graphite or CuSO_4_ at 9.18 MPA.

The effect of graphite ratio on the tensile strength of composites was found to be statistically not significant, which means the nonwoven reinforced composites mentioned in this paper were functionalized to be electrically conductive without sacrificing their mechanical properties.

The effect of the waste ratio on the tensile strength of composites was statistically significant. In general, it is observed that tensile strength increased with decreasing waste ratio since virgin fibers have better strength and longer lengths compared to recycled fibers, resulting in better bonding in the nonwoven structure.

The effects of thickness and basis weight of composites on the tensile strength of composites were statistically significant. It can be shown that an increase in the thickness and basis weight of composites causes an increase in tensile strength due to more fibers and resin in the structure.

The effect of the thickness and basis weight of reinforcement material on the tensile strength of composites is statistically significant. It can be commented that tensile maximum stress increased with increasing reinforcement thickness and basis weight due to more fibers and resin in the structure.

Reinforcement air permeability has a statistically significant effect on the tensile strength of composites. Tensile maximum stress increased with decreasing air permeability. This can be explained by the higher fiber density of the samples with lower air permeability.

#### 3.5.2. Tensile Max. Elongation

Results of ANOVA tests have shown that waste fiber ratio, thickness, basis weight, reinforcement material’s thickness, reinforcement material’s basis weight, and reinforcement material’s air permeability have statistically significant effects, whereas graphite and CuSO_4_ ratio did not have a statistically significant effect on the tensile max. elongation of composites within a 95% confidence interval.

**Figure 23:**
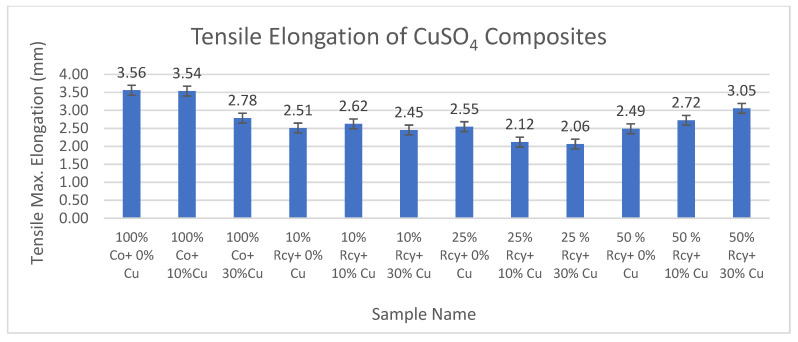
Tensile max. elongation of CuSO_4_ added composites.

**Figure 24:**
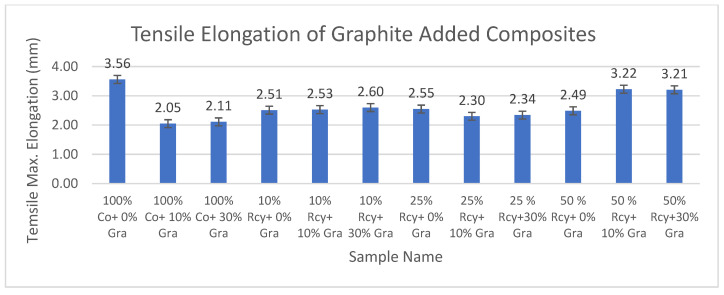
Tensile max. elongation of graphite added composites.

Results of tensile max. elongation of the CuSO_4_ added nonwoven reinforced composites are given in Figure 23, and results of graphite-added nonwoven reinforced composites are given in Figure 24.

The effects of graphite ratio and CuSO_4_ ratio on the tensile max. elongation of composites were found to be statistically not significant. Thus, it is illustrated in the above figures that adding conductive particles did not reduce the tensile maximum elongation values significantly.

The effect of waste ratio on the tensile max. elongation of composites was found to be statistically significant. In general, it can be commented that tensile modulus increased with increasing waste ratio. This is an expected result because tensile strength increased with decreasing waste ratio since virgin fibers have better strength and longer lengths compared to recycled fibers, resulting in better bonding in the nonwoven structure.

The effects of thickness and basis weight of composites on the tensile max. elongation of composites are statistically significant. In general, it can be said that an increase in the thickness and basis weight of composites causes an increase in tensile maximum elongation.

The effects of reinforcement material’s thickness and basis weight on the tensile max. elongation of composites were found to be statistically significant. As a general trend, it can be commented that tensile maximum elongation decreased with increasing reinforcement thickness and basis weight.

Reinforcement material’s air permeability had a statistically significant effect on the tensile max. elongation of composites, and it was seen that the tensile maximum elongation of composites increased with decreasing air permeability.

### 3.6. Results of Bending Tests

#### 3.6.1. Bending Strength

Results of statistical analysis have shown that waste ratio, CuSO_4_ ratio, basis weight, reinforcement material’s thickness, reinforcement material’s basis weight, and reinforcement material’s air permeability have statistically significant effects on the bending strength of nonwoven reinforced composites, whereas graphite ratio, thickness, and woven orientation did not have a statistically significant effect within a 95% confidence interval.

**Figure 25:**
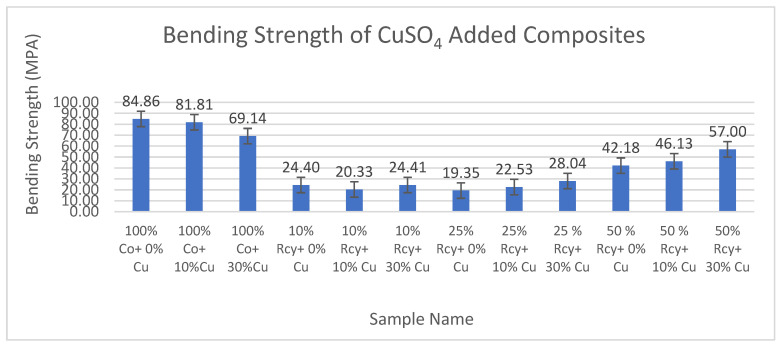
Bending strength of CuSO_4_ added composites.

**Figure 26:**
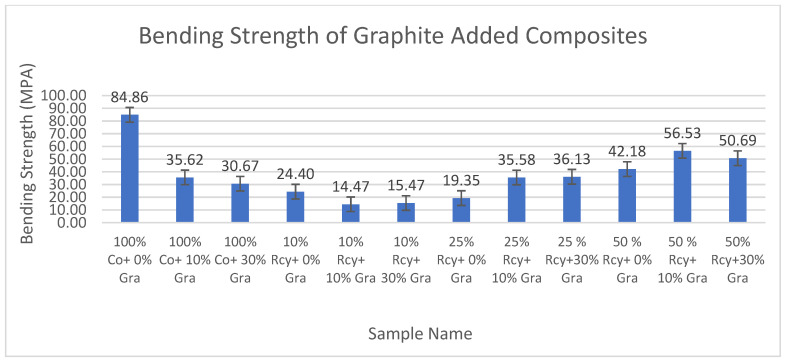
Bending strength of graphite-added composites.

Results of bending strength tests of the CuSO_4_ added nonwoven reinforced composites are given in Figure 25, and results of graphite-added nonwoven reinforced composites are given in Figure 26.

It was found that the effect of the graphite ratio on the bending strength of composites was statistically not significant. Thus, it can be concluded that adding graphite to the structure did not reduce the bending strength values. The highest bending strength values were obtained from 100% cotton composites that did not contain any conductive particles, at 84.86 MPA.

The effect of the CuSO_4_ ratio on the bending strength of composites was statistically significant. The highest bending strength values were obtained samples which does not contain CuSO_4_.

Results have shown that the effect of waste ratio on the bending strength of composites was statistically significant. The difference between 0% and 10% waste ratios were found to be statistically not significant. Considering the results of 25% and 50% wastes, it is observed that an increase in waste ratio causes a decrease in bending strength.

The effect of the basis weight of composites on the bending maximum stress of composites is statistically significant. It can be commented on in general that bending maximum stress decreased with decreasing basis weight. The effect of the thickness of composites on the bending maximum stress of composites is statistically insignificant.

It was also found that reinforcement material’s air permeability has a statistically significant effect on the bending maximum stress of composites, and in general, it is shown that the bending strength of composites increases with increasing air permeability.

#### 3.6.2. Bending Max. Elongation

Results of statistical analysis have shown that thickness, basis weight, reinforcement material’s thickness, reinforcement material’s basis weight, and reinforcement material’s air permeability have statistically significant effects on the bending max. elongation of composites, whereas graphite ratio, CuSO_4_ ratio, and waste fiber ratio did not have a statistically significant effect within a 95% confidence interval.

**Figure 27:**
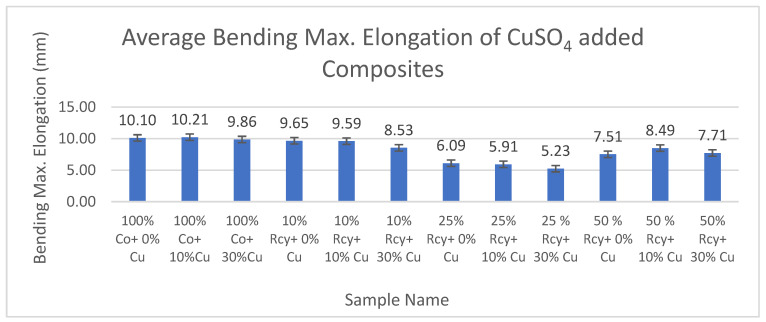
Bending max. elongation of CuSO_4_ added composites.

**Figure 28:**
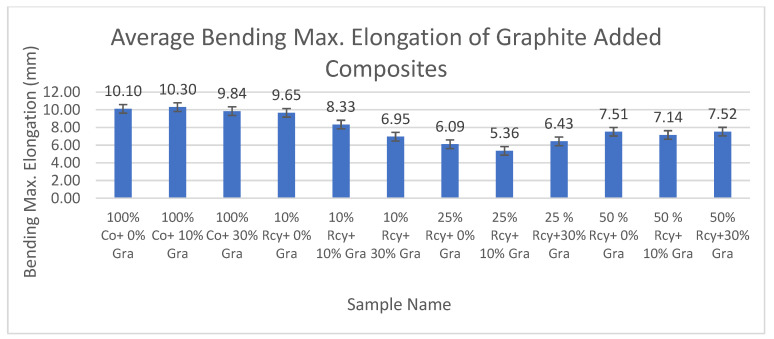
Bending the max elongation values of graphite-added composites.

Results of bending max. elongation of the CuSO_4_ added nonwoven reinforced composites are given in Figure 27, and results of graphite-added nonwoven reinforced composites are given in Figure 28.

The effects of waste ratio, graphite ratio, and CuSO_4_ ratio on the bending maximum elongation of composites were not statistically significant. On the other hand, the effect of thickness and basis weight of composites and reinforcement material on bending max. elongation was found to be statistically significant. It can be said that bending max. elongation increased with decreasing thickness and basis weight of the composites and reinforcement material, as well as air permeability.

The effect of thickness and basis weight of reinforcement material on bending maximum elongation of composites is statistically significant. In general, it can be commented that bending maximum elongation increased with thickness and basis weight of the reinforcement material.

Reinforcement material’s air permeability had a statistically significant effect on bending maximum elongation of composites, and in general, it can be noted that bending maximum elongation of composites increased with decreasing air permeability.

## 4. Conclusions

In this paper, the average electromagnetic shielding value of CuSO_4_ added nonwoven reinforced composites was 18.69 dB, and the average electromagnetic shielding value of graphite-added nonwoven reinforced composites was 18.70 dB.

The maximum electromagnetic shielding effectiveness of CuSO_4_ added nonwoven reinforced material was 31.43 dB at 6 GHz, which was obtained from a sample including a 10% waste fiber ratio and a 30% CuSO_4_ ratio. For graphite-added composites, the highest value, 29.90 dB, was obtained at 6 GHz from the sample, including a 50% waste ratio and a 30% graphite ratio. The lowest average electromagnetic shielding effectiveness value was obtained at 14.39 dB at 2 GHz, and the highest average electromagnetic shielding effectiveness value was obtained at 26.21 dB at 6 GHz. The composites developed in this study have excellent shielding effectiveness for daily use and middle shielding effectiveness for professional use, exceeding the acceptable range for industrial and commercial applications of 20 dB [42,48]. The developed composites possess properties like light weight, corrosion resistance, and low cost, besides electromagnetic shielding effectiveness, which make them good candidates to replace metals in some applications where 31 dB shielding effectiveness is sufficient.

In future studies, specific application areas of light-weight electromagnetic shielding composites, such as household devices, electronic industry, and building and construction industries, can be selected and investigated based on the results obtained in this paper.

## Figures and Tables

**Table 1:** Some of the related studies about electromagnetic composites produced with nonwoven reinforcement are in the literature.

Writer/Year	Reinforcement	Matrix	Method	EMSE (dB)	Difference of Our Study
Hu et al., 2023 [27]	Cupper Coated PET Nonwoven	Polyamid + Carbon Fiber	Lamination	88.6	Recycled fibers, Conductive Particles, Matrix, Productionmethod
Sedighi et al., 2022 [28]	PET Nonwoven	Polyaniline (PANI) + rGO and Fe_3_O_4_ nanopowders	Layer-by-layer	76.6	Recycled Fibers, Conductive particles type, Matrix, Production method
Liu et al., 2019 [29]	Fiber-Welded Nonwoven + Graphene Oxide (GO) Sheets	Epoxy	Resin Transfer Molding	65	Recycled fibers. Production of Reinforcement, Matrix, Composite production methodMethod
Gao et al., 2021 [30]	Cotton Nonwoven	Polydopamine (PDA) + Ag nanoparticles	Coating	110	Recycled fibers, Type of conductive particles, Matrix, Production Method
Maity and Chatterjee, 2018 [31]	Polyester Nonwoven	Polypyrrole (PPy)	Coating	20.07	Recycled Fibers, Conductive Polymer Matrix, Production Method
Zhao et al., 2016 [32]	PET Nonwoven	Acrylic Adhesive + Carbon Fiber	Layer-by-layer	60.49	Recycled Fibers, Conductice Fibers, Matrix, Method
Fan et al., 2021 [9]	PET Nonwoven + Graphene (GE) nanosheets	Polyvinylidenefluoride (PVDF)	Coating	31.2	Recycled Fibers, Matrix, Production methodMethod
Herrera et al., 2018 [33]	Nonwoven+ MWCNT filled carbon nanofiber	Nanoreinforced Polymer (PP) Composite Sheets (NRPCS)	Compression-molded	17	Recycled fibers, Conductive Particles, Matrix, Production Method
Lu et al., 2019 [34]	Carbon Fiber Nonwoven (CFNW) + Polyaniline (PANI)	Epoxy	Screen coating	61	Recycled fibers, Matrix, ProductionMethod
Ren et al., 2020 [35]	Ag Coated Nonwoven	Waterborne Polyurethane (WPU) Film + Ag Particles	Casting	72.5	Production of Reinforcement, Conductive Particles Matrix, Method
Ali et al., 2022 [12]	Recycled Kevlar Nonwoven	Epoxy + Carbon Particles	Hand Lay Up	35.6	Reinforcement material and Matrix,
Lin et al., 2022 [36]	Carbon fiber woven fabric and nylon spacer fabric	Low melting point PES nonwoven	Lamination	65	Nonwoven reinforcement with recycled fibers

**Table 2:** Parameters of the needle punching process.

Parameter	Value
Needle depth (cm)	0.8
Speed of the conveyor belt of needling and wind up (m/min)	4
Speed of the conveyor belt of cross lapper (m/min)	2

**Table 3:** Composition of Nonwoven Reinforced Composites.

Sample Name	Reinforcement	Resin
100% Co + 0% Cu	100% Cotton	100% PES
100% Co + 10% Cu	100% Cotton	90% PES + 10% CuSO_4_
100% Co + 30% Cu	100% Cotton	70% PES + 30% CuSO_4_
10% Rcy + 0% Cu	10% Recycled fiber + 90% Cotton	100% PES
10% Rcy + 10% Cu	10% Recycled fiber + 90% Cotton	90% PES + 10% CuSO_4_
10% Rcy + 30% Cu	10% Recycled fiber + 90% Cotton	70% PES + 30% CuSO_4_
25% Rcy + 0% Cu	25% Recycled fiber + 75% Cotton	100% PES
25% Rcy + 10% Cu	25% Recycled fiber + 75% Cotton	90% PES + 10% CuSO_4_
25% Rcy + 30% Cu	25% Recycled fiber + 75% Cotton	70% PES + 30% CuSO_4_
50% Rcy + 0% Cu	50% Recycled fiber + 50% Cotton	100% PES
50% Rcy + 10% Cu	50% Recycled fiber + 50% Cotton	90% PES + 10% CuSO_4_
50% Rcy + 30% Cu	50% Recycled fiber + 50% Cotton	70% PES + 30% CuSO_4_
100% Co + 0% Gra	100% Cotton	100% PES
100% Co + 10% Gra	100% Cotton	90% PES + 10% Graphite
100% Co + 30% Gra	100% Cotton	70% PES + 30% Graphite
10% Rcy + 0% Gra	10% Recycled fiber + 90% Cotton	100% PES
10% Rcy + 10% Gra	10% Recycled fiber + 90% Cotton	90% PES + 10% Graphite
10% Rcy + 30% Gra	10% Recycled fiber + 90% Cotton	70% PES + 30% Graphite
25% Rcy + 0% Gra	25% Recycled fiber + 75% Cotton	100% PES
25% Rcy + 10% Gra	25% Recycled fiber + 75% Cotton	90% PES + 10% Graphite
25% Rcy + 30% Gra	25% Recycled fiber + 75% Cotton	70% PES + 30% Graphite
50% Rcy + 0% Gra	50% Recycled fiber + 50% Cotton	100% PES
50% Rcy + 10% Gra	50% Recycled fiber + 50% Cotton	90% PES + 10% Graphite
50% Rcy + 30% Gra	50% Recycled fiber + 50% Cotton	70% PES + 30% Graphite

## Data Availability

The data presented in this study are available on request from the corresponding author.

## References

[1] Kadoğlu H., Duran D. (2015). Electromagnetic shielding characterization of conductive woven fabrics produced with silver-containing yarns. Text. Res. J..

[2] Šafářová V., Militký J. (2014). Electromagnetic shielding properties of woven fabrics made from high-performance fibers. Text. Res. J..

[3] Yılmaz R. (2014). Elektromanyetik Kalkanlama Özelliği Olan Malzemeler. Ejovoc (Electron. J. Vocat. Coll.).

[4] (2011). Parliamentary Assambly, Committee on the Environment, Agriculture and Local and Regional Affairs. The Potential Dangers of Electromagnetic Fields and Their Effect on the Environment, CE, Strasbourg. https://assembly.coe.int/nw/xml/XRef/Xref-XML2HTML-en.asp?fileid=17994.

[5] Duran D. (2011). A Research on Application Possibilities of Textile Materials in Electromagnetic Shielding Applications. Ph.D. Thesis.

[6] Oğul H., Polat H., Akman F., Kaçal M.R., Dilsiz K., Bulut F., Agar O. (2022). Gamma and Neutron Shielding Parameters of Polyester-based composites reinforced with boron and tin nanopowders. Radiat. Phys. Chem..

[7] Liu P., Ng V.M.H., Yao Z., Zhou J., Lei Y., Yang Z., Lv H., Kong L.B. (2017). Facile Synthesis and Hierarchical Assembly of Flowerlike NiO Structures with Enhanced Dielectric and Microwave Absorption Properties. Appl. Mater. Interfaces.

[8] Nan Z., Wei W., Lin Z., Chang J., Hao Y. (2023). Flexible Nanocomposite Conductors for Electromagnetic Interference Shielding. Nano-Micro Lett..

[9] Fan Z., Liu R., Cheng X. (2021). Preparation and Characterization of Electromagnetic Shielding Composites Based on Graphene-Nanosheets-Loaded Nonwoven Fabric. Coatings.

[10] Lu L., Xing D., Teh K.S., Liu H., Xie Y., Liu X., Tang Y. (2017). Structural effects in a composite nonwoven fabric on EMI shielding. Mater. Des..

[11] Wong K.H., Pickering S.J., Rudd C.D. (2010). Recycled carbon fibre reinforced polymer composite for electromagnetic interference shielding. Compos. Part A Appl. Sci. Manuf..

[12] Ali A., Hussain F., Tahir M.F., Ali M., Zaman Khan M., Tomková B., Militky J., Noman M.T., Azeem M. (2022). Fabrication of Conductive, High Strength and Electromagnetic Interference (EMI) Shielded Green Composites Based on Waste Materials. Polymers.

[13] Yadong X., Yaqi Y., Ding-Xiang Y., Hongji D., Guizhe Z., Yaqing L. (2019). Flexible and conductive polyurethane composites for electromagnetic shielding and printable circuit. Chem. Eng. J..

[14] Liang L., Yao C., Yan X., Feng Y., Hao X., Zhou B., Wang Y., Ma J., Liu C., Shen C. (2021). High-efficiency electromagnetic interference shielding capability of magnetic Ti3C2Tx MXene/CNT composite film. Mater. Chem. A.

[15] Keeling J.L. (2017). Graphite: Properties, uses and Australian resources. Mesa J..

[16] Derek P.C., Davenport W.G. (1980). Densities, Electrical Conductivities and Viscosities of CuSO4/H2SO4 Solutions in the Range of Modern Electrorefining and Electrowinning Electrolytes. Metall. Trans. B.

[17] Patnaik P.K., Swain P.T.R., Mishra S.K., Purohit A., Biswas S. (2020). Recent developments on characterization of needle-punched nonwoven fabric reinforced polymer composites—A review. Mater. Today Proc..

[18] Rawal A., Majumdar A., Anand S., Shah T. (2009). Predicting the properties of needlepunched nonwovens using artificial neural network. J. Appl. Polym. Sci..

[19] Duran D. (2016). A Research on Thermal Insulatıon Propertıes of Nonwovens Produced With Recycled Jute And Wool Fıbres. Text. Appar..

[20] Cheng J., Li J., Xiong Y., Zhang H., Raza H., Ullah S., Wu J., Zheng G., Cao Q., Zhang D. (2022). Recent Advances in Design Strategies and Multifunctionality of Flexible Electromagnetic Interference Shielding Materials. Nano-Micro Lett..

[21] Atay M. (2022). An Investigation on the Production of Non-Woven Textile Surface Reinforced Electromagnetic Shielding Composites from Recycled Fibers. Master’s Thesis.

[22] Pakdel E., Kashi S., Baum T., Usman K.A.S., Razal J.M., Varley R., Wang X. (2021). Carbon fibre waste recycling into hybrid nonwovens for electromagnetic interference shielding and sound absorption. J. Clean. Prod..

[23] Zeng Z., Jin H., Chen M., Li W., Zhou L., Xue X., Zhang Z. (2017). Microstructure design of lightweight, fexible, and high electromagnetic shielding porous multiwalled carbon nanotube/polymer composites. Small.

[24] Omrani F., Soulat D., Ferreira M., Wang P. (2019). Effects of needle punching process and structural parameters on mechanical behavior of flax nonwovens preforms. Adv. Aircr. Spacecr. Sci..

[25] Wilson A. (2010). The formation of dry, wet, spunlaid and other types of nonwovens. Applications of Nonwovens in Technical Textiles.

[26] Chen X., Chen L., Zhang C., Song L., Zhang D. (2016). Three-dimensional needle-punching for composites—A review. Compos. Part A.

[27] Hu S., Wang D., Venkataraman M., Křemenáková D., Militký J., Yang K. (2023). Enhanced electromagnetic shielding of lightweight copper-coated nonwoven laminate with carbon filament reinforcement. J. Eng. Fibers Fabr..

[28] Sedighi A., Naderi M., Brycki B. (2023). Wearable nonwoven fabric decorated with Fe3O4/rGO/PANI/Ni-P for efficient electromagnetic interference shielding. J. Alloys Compd..

[29] Liu L., Wang H., Shan M., Jiang Y., Zhang X., Xu Z. (2019). Lightweight sandwich fiber-welded foam-like nonwoven fabrics/graphene composites for electromagnetic shielding. Mater. Chem. Phys..

[30] Gao Y.N., Wang Y., Yue T.N., Weng Y.X., Wang M. (2021). Multifunctional cotton non-woven fabrics coated with silver nanoparticles and polymers for antibacterial, superhydrophobic and high performance microwave shielding. J. Colloid Interface Sci..

[31] Maity S., Chatterjee A. (2018). Polypyrrole functionalized polyester needlepunched nonwoven fabrics for electro-magnetic interference shielding. Polym. Compos..

[32] Zhao X., Fu J., Wang H. (2016). The electromagnetic interference shielding performance of continuous carbon fiber composites with different arrangements. J. Ind. Text..

[33] Ramírez-Herrera C.A., Gonzalez H., Torre F., Benitez L., Cabañas-Moreno J.G., Lozano K. (2019). Electrical Properties and Electromagnetic Interference Shielding Effectiveness of Interlayered Systems Composed by Carbon Nanotube Filled Carbon Nanofiber Mats and Polymer Composites. Nanomaterials.

[34] Lu H., Liao B., Wang H., Xu Z., Li N., Liu L., Wu N. (2019). Electromagnetic shielding of ultrathin, lightweight and strong nonwoven composites decorated by a bandage-style interlaced layer electropolymerized with polyaniline. J. Mater. Sci. Mater. Electron..

[35] Ren W., Zhu H., Yang Y., Chen Y., Duan H., Zhao G., Liu Y. (2020). Flexible and robust silver coated non-woven fabric reinforced waterborne polyurethane films for ultra-efficient electromagnetic shielding. Compos. Part B Eng..

[36] Lin J.H., Hsu P.W., Huang C.H., Lai M.F., Shiu B.C., Lou C.W. (2022). A Study on Carbon Fiber Composites with Low-Melting-Point Polyester Nonwoven Fabric Reinforcement: A Highly Effective Electromagnetic Wave Shield Textile Material. Polymers.

[37] Ütebay B., Çelik P., Çay A. (2019). Effects of cotton textile waste properties on recycled fibre quality. J. Clean. Prod..

[38] Araujo E.M., Araujo K.D., Pereira O.D., Ribeiro P.C., Melo T.J.A. (2006). Fiberglass Wastes/Polyester Resin Composites: Mechanical Properties and Water Sorption. Polím. Ciênc. E Tecnol..

[39] (2022). Standard Test Method for Through-Thickness “Flatwise” Tensile Strength and Elastic Modulus of a Fiber-Reinforced Polymer Matrix Composite Material.

[40] (2020). Standard Test Methods for Density and Specific Gravity (Relative Density) of Plastics by Displacement.

[41] (2005). Anechoic Chambers-Part-1: Shield Attenuation Measurement.

[42] (2005). Test Method of Specified Requirements of Electromagnetic Shielding Textiles.

[43] (2007). Standard Test Methods for DC Resistance or Conductance of Insulating Materials.

[44] (2014). Standard Test Method for Tensile Properties of Polymer Matrix Composite Materials.

[45] (2017). Standard Test Methods for Flexural Properties of Unreinforced and Reinforced Plastics and Electrical Insulating Materials.

[46] Liu X., Li Y., Sun X., Tang W., Deng G., Liu Y., Shui J. (2021). Of/on switchable smart electromagnetic interference shielding aerogel. Matter.

[47] Tserpers K., Tzatzadakis V., Bachmann J. (2020). Electrical Conductivity and Electromagnetic Shielding Effectiveness of Bio-Composites. J. Compos. Sci..

[48] Afilipoaei C., Draghicescu H.T. A Review over Electromagnetic Shielding Effectiveness of Composite Materials. Proceedings of the 14th International Conference on Interdisciplinarity in Engineering—INTER-ENG 2020.

